# Influence of different control strategies on muscle activation patterns in trunk muscles

**DOI:** 10.14814/phy2.12229

**Published:** 2014-12-11

**Authors:** Laura Hansen, Christoph Anders

**Affiliations:** 1 Clinic for Trauma, Hand and Reconstructive Surgery, Division of Motor Research, Pathophysiology and Biomechanics, Jena University Hospital, Jena, Germany

**Keywords:** Abdominal muscles, back muscles, control strategy, SEMG (surface electromyography)

## Abstract

Adequate training of the trunk muscles is essential to prevent low back pain. Although sit‐ups are simple to perform, the perceived high effort is the reason why training the abdominal muscles is seldom continued over a longer period of time. It is well known that the abdominal muscles are inferior to the back muscles in terms of force, but this cannot explain the extreme difference in perceived effort between trunk flexion and extension tasks. Therefore, this study was aimed at the identification of control strategy influences on the muscular stress level. Thirty‐nine subjects were investigated. The performed tasks were restricted to the sagittal plane and were implemented with simulated and realized tilt angles. Subjects were investigated in an upright position with their lower bodies fixed and their upper bodies free. Posture‐controlled tasks involved graded forward and backward tilting, while force‐controlled tasks involved the application of force based on a virtual tilt angle. The Surface EMG (SEMG) was taken from five trunk muscles on both sides. Control strategies seemed to have no systematic influence on the SEMG amplitudes of the back muscles. In contrast, the abdominal muscles exhibited significantly higher stress levels under posture‐controlled conditions without relevantly increasing antagonistic co‐activation of back muscles. The abdominal muscles' relative differences ranged from an average of 20% for the external oblique abdominal muscle to approximately 40% for the rectus abdominal muscle. The perceived high effort expended during sit‐ups can now be explained by the posture‐controlled contractions that are required.

## Abstract (precis)

We performed posture and force‐controlled trunk flexion and extension tasks at varying submaximal load levels while remaining in an upright posture. Abdominal muscles exhibited up to 40% higher effort during posture‐controlled situations compared to the force‐controlled situations, but, in contrast, back muscles were completely unaffected from the applied control strategy. Therefore, our back muscles can be used universally under the aspect of control strategies, but posture‐controlled tasks of abdominal muscles, that is, sit‐ups require significantly higher effort.

## Introduction

Adequate coordination of the entirety of the trunk muscles is essential for ensuring the stability and mobility of the spine (Gardner‐Morse and Stokes 1998; McGill et al. 2003). Therefore, the study of trunk muscles by means of Surface EMG (SEMG) has been the focus of research for years (Hides et al. 1994; Hodges and Richardson 1996; Kankaanpää et al. 1996; Magnusson et al. 1996; Leinonen et al. 2003). The results of studies investigating the function of trunk muscles demonstrated that disruptions of trunk muscle coordination are associated with lower back pain (Panjabi 2003).

These fundamental scientific findings have entered mainstream consciousness: a healthy back is no longer considered to be the responsibility of only the back muscles; rather, abdominal muscle function is now perceived to be equally important. Consequently, the question rose of what training methods are adequate to maintain this function.

Although sit‐ups do not improve all aspects of the coordination behavior of abdominal muscles (Tsao and Hodges 2007), they are often implemented to prevent back pain. Because they are simple to do, this exercise is also often recommended for rehabilitation. Usually; however, when performing sit‐ups the perception of an extremely high effort quickly takes the foreground; therefore, the abdominal muscles training is seldom continued over a long period of time if not forced from outside factors. This high effort is supported by EMG data showing that the rectus abdominis muscle reached up to 80% of its maximal activity level during sit‐ups (Cordo et al. 2003; Burden and Redmond 2013).

Because of their significantly smaller physiological cross‐sectional area, the performance of abdominal muscles is inferior to the back muscles in terms of force (Keller and Roy 2002). Thus, it is, at first, not surprising that back extension exercises are associated with significantly less effort than sit‐ ups. Nevertheless, the isometric force difference (extension/flexion ratio: male = 1.13; female = 1.7; Keller and Roy 2002) cannot fully explain the extreme difference in perceived effort that occurs between free trunk flexion and extension tasks, that is, during situations controlling postures or performing movements, rather than developing forces against a fixed resistance. Although everyone would agree with this perception, data required to scientifically support this effect are not available in the literature. This raises the question of whether the way an exercise is performed can contribute to the observed effort differences. Of interest in this context is the perceived effort of the isometric muscle contractions against resistance (force‐controlled) compared to the stabilization of the upper body against gravity (posture‐controlled).

To objectify the perceived effort, the muscular stress level can be used. This describes the effect of a particular load on the muscular system, which can be assessed by SEMG amplitude traces. Furthermore, Anders et al. (2008) found that the muscles of the trunk demonstrate different SEMG amplitude–force relationships. While the back muscles are characterized by a linear SEMG increase with increasing force, the abdominal muscles have an S‐shaped curve signature.

These findings raise the possibility that the higher perceived effort associated with using abdominal muscles is caused merely by the nonlinearity of the amplitude–force relationship. Thereby, the muscular stress level increases disproportionately once a certain level of force is reached. Minor differences in force level thus lead to large changes of the amplitude, and thus to the perceived effort.

Whether the distinctive amplitude–force relationship underlies the different perceived effort, or whether this could indeed be due to the type of motor control strategy was investigated in the present study. We hypothesized, that the posture‐controlled strategy would require higher effort for the abdominal but not for the back muscles, if compared with the force‐controlled strategy.

## Methods

The study included 39 subjects: 19 women and 20 men (their anthropometric data are presented in Table 1). The subjects were clinically healthy in terms of their medical histories and cardiopulmonary statuses, and had no prior injuries to the musculoskeletal system. Participation was voluntary. Informed written consent was obtained from each volunteer. The data presented here are part of a larger study that was approved by the local ethics committee of the Jena University (3021‐01/11) and therefore comply with international ethical standards.

**Table 1.** Test subjects.

	Age (years)	Weight (kg)	Height (cm)	BMI (kg/m²)
All				
Median	24.0	65.1	174	22.3
Upper quartile	2.0	8.1	6.0	1.3
Lower quartile	1.0	3.5	4.0	1.5
Women				
Median	24.0	60.5	170	21.4
Upper quartile	1.0	3.8	3.0	1.1
Lower quartile	1.0	2.5	2.0	0.7
Men				
Median	25.5	72.9	180	22.6
Upper quartile	2.8	3.3	1.3	1.4
Lower quartile	1.8	5.5	5.3	0.9
*P*‐value	0.077	0.001	0.001	0.1

BMI, body mass index.

### Test device

**Figure 1.**
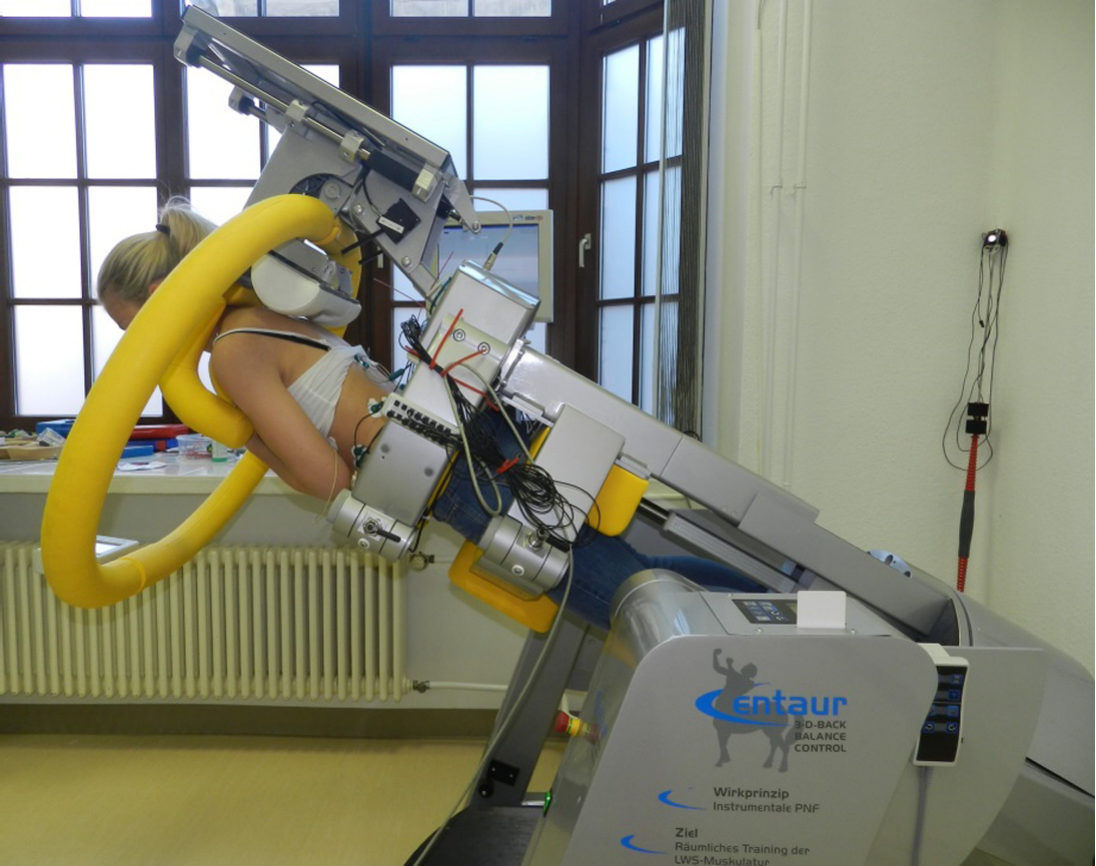
Subject performing a posture‐controlled position at a 60° forward tilt angle. Arms are held crossed over the chest (working posture). The upper body is held along the body axis to keep the control point on the display in the center of the crosshair.

The tests were conducted in a computerized testing and training device (CTT CENTAUR, BfMC, Leipzig, Germany), a multi‐functional device that provides for basic physiological testing of the trunk muscles, among others. The lower body up to the hips of the subject was immobilized to the CENTAUR, while the upper body had freedom of mobility. By setting different tilt angles, the body position in the gravitational field of the earth changes (Fig. 1). Because full mobility of the upper body is given, stabilization can occur along the longitudinal axis of the body. An open harness, equipped with strain gauges, plus a display at shoulder level serves as a biofeedback system. As long as the investigated subject remains in a neutrally aligned position with the lower body and relieves the strain gauges from the upper body weight, no force is applied to the harness. Consequently, the control point on the display remains in the center of the crosshair.

The aim of this study was to investigate whether the two different control strategies influence on the EMG activity of the trunk muscles. The performed tasks were restricted to the sagittal plane and were implemented with simulated and realized tilt angles of 5, 10, 20, 30, 45, 60, and 90°. A total of 28 individual tests were conducted, divided into two groups: posture‐controlled tasks and force‐controlled tasks.

## Experimental Procedures

At the beginning, the subjects were brought to a horizontal position by a 90° forward tilt; their upper body weight was subsequently measured by the harness strain gauges. To check for relaxation of the back muscles, SEMG traces were registered and flags were used to indicate when residual contractions were present. To obtain trustworthy values, this procedure was repeated three times and the highest value was then used.

In the posture‐controlled test, the CENTAUR was gradually tilted forwards or backwards. Therefore, at a tilt angle of 90° the entire upper body weight (100%) was applied as the force moment, while at the other tilt angles the force applied corresponded to 9, 17, 34, 50, 71, and 87% of upper body weight. At each tilt angle the subjects were asked to relieve the harness of the body weight to keep the control point in the center of the crosshair. By adopting the neutrally aligned position the respective percentages of the upper body weight had to be supported.

In the force‐controlled test, subjects remained in an upright position and were requested to apply the harness forces corresponding to the fraction of the upper body weight at each of the selected tilt angles. The respective forces were visualized by displaying a proportionally deviated control point (on an external screen), corresponding to each selected force. To fulfill the task the control point had to be brought back to zero by applying the required force to the harness via contraction of the trunk muscles. This was also controlled and corrected if necessary by the investigator.

To exclude systematic order effects, an individually randomized design of the respective tests was created for each subject, which included both performance profiles. The posture‐controlled 90° tilts, the element assumed to be most difficult, were always performed at the beginning of the test. Throughout the entire test, the subjects remained in the so‐called ‘working posture’ (arms crossed over chest) to exclude effects due to varying arm positions. After completing the tests, the subjects were questioned separately about the forward and backward tilt tests to determine which control strategy was perceived as more exhausting.

### SEMG measurement and analysis

For each of the 28 tests, the SEMG was measured by applying a bipolar montage from a total of five trunk muscles simultaneously on both sides of the body. The electrodes were applied according to standard protocol and according to the specifications in Table 2 by the same experienced examiner (CA). Circular Ag‐AgCl solid‐gel electrodes were used (H93SG, Covidien, Neustadt, Germany) with a surface area of 2 cm². The distance between the electrodes was 2.5 cm. Simultaneously with the SEMG channels, the force channels in the X (sagittal plane) and Y (frontal plane) direction and the rotational and/or tilt angle of the CENTAUR were recorded. The force channels provided data in Newtons while the others provided the corresponding angles in degrees. In addition, cardiac traces were recorded for subsequent electrocardiographical (ECG) artifact elimination. The measured values were amplified by 1000 (Biovision, Werheim, Germany). Digitization of the raw signals was performed using an analog‐to‐digital (A/D) converter (Tower of Measurement (DeMeTec, Lützelwiesen, Germany), A/D rate 2000/s; 24 bit resolution at ±5 V, anti‐aliasing filter at 1000 Hz). The data were collected (GJB, Langewiesen, Germany) for later processing and stored on a hard drive.

**Table 2.** SEMG electrode positions.

Muscle	Electrode localization/orientation
Rectus abdominis (RA)	Caudal electrode at navel height, 4 cm from center, vertical
Obliquus internus (OI)	Medial inguinal ligament, at anterior superior iliac spine height, horizontal
Obliquus externus (OE)	Cranial electrode directly below lowest point of the costal arch, on line from there to contralateral pubic tubercle
Multifidus (MF)	Caudal electrode at L5 height, 1 cm medial and parallel to line between posterior superior iliac spine line and L1
Longissimus (LO)	Caudal electrode at L1 height, over palpable bulge of muscle (approx. 2 fingers lateral from midline), vertical
ECG	Along heart axis, above heart

ECG, Electrocardiogram.

Further processing of the raw data was performed using the computer software Matlab (The Mathworks, Natick, MA) and ATISAPro (GJB). To eliminate the ECG signals from the SEMG data, 100 msec after the detection of each R wave, an interval of 400 msec was used for further processing. Depending on the number of QRS complexes within the measurement period, this resulted in a total number of 9–15 signal regions that could be evaluated per recorded load situation. From these amplitude values the mean root mean square (RMS) values were calculated in the frequency range between 20–400 Hz by averaging all respective regions.

### Statistics

The RMS amplitude differences between corresponding load levels of both control regimes were calculated so that disparities between the two control strategies could be identified directly. In order to account for different amplitude levels relative amplitude differences were determined as well. The calculated differences appear as positive values if higher values occurred during the posture‐controlled situation, negative values appear for the opposite case. Both parameters were tested according to deviations from zero, that is, systematically different values between both situations were tested using the student's *t*‐Test for paired samples, separately for every muscle and load level.

Because the parameters showed characteristics of a normal distribution separately for every muscle, a repeated measures analysis of variance (ANOVA) was conducted to determine whether control strategies and load levels had an influence on our findings. Consequently, the ANOVA of the RMS amplitudes (control (2) × load (7)), and, to test for load dependencies according to the observed deviations, the relative amplitude differences (load (7)), were calculated. All calculations were conducted separately for the main and opposite force directions.

For the post hoc test, the student's *t*‐tests for paired samples including Bonferroni correction to control for type 1 error was applied according to the load levels. The internationally accepted level of 5% was defined as the statistical significance level.

## Results

The ANOVA of the RMS values for the main force directions supports the hypothesis that control strategies significantly influence the abdominal but not the back muscles (Table 3). For the opposite force directions, except for the external oblique muscle (OE), the RMS values were also significantly influenced by the control strategy. Likewise, the tilt angle had significant influence on all muscles, independent of the force direction.

**Table 3.** ANOVA results (*P* values) for the RMS SEMG values for posture and force‐controlled tasks.

	Muscle	Control	Angle	Angle × Control
Main force direction	RA	<0.001	<0.001	<0.001
	OI	<0.001	<0.001	<0.001
	OE	<0.001	<0.001	<0.001
	MF	0.516	<0.001	<0.001
	LO	0.268	<0.001	0.483
Opposite force direction	RA	0.002	<0.001	0.135
	OI	<0.001	<0.001	<0.001
	OE	0.578	<0.001	0.001
	MF	0.008	<0.001	<0.001
	LO	0.033	<0.001	<0.001

Concerning the relative amplitude differences for the main force directions, the load dependent influences were found to affect all muscles except longissimus muscle (LO) (Table 4). The relative differences of all abdominal muscles mainly differed for low load levels. Only multifidus muscle (MF) displayed multiple contrasts as its values changed between slightly positive and negative values.

**Table 4.** ANOVA together with significant post hoc test results (including Bonferroni correction) for relative differences (*P* values).

			5°	10°	20°	30°	45°	60°
Main force direction	RA <0.001	10°	<0.001					
		20°	<0.001					
		30°	<0.001					
		45°	<0.001					
		60°	<0.001					
		90°	<0.001					0.002
	OI 0.002	10°						
		20°						
		30°						
		45°	0.023					
		60°						
		90°						
	OE <0.001	10°	0.018					
		20°		<0.001				
		30°	0.041	<0.001				
		45°	0.026	<0.001				
		60°	0.007	<0.001				
		90°		<0.001				
	MF <0.001	10°						
		20°						
		30°		0.006	0.018			
		45°	0.040	<0.001	<0.001			
		60°	0.022	0.010	0.010			
		90°					0.030	0.010
	LO 0.484	10°						
		20°						
		30°						
		45°						
		60°						
		90°						
Opposite force direction	RA 0.476	10°						
		20°						
		30°						
		45°						
		60°						
		90°						
	OI 0.027	10°						
		20°						
		30°						
		45°						
		60°		0.039				
		90°						
	OE <0.001	10°						
		20°						
		30°						
		45°	0.003	0.005				
		60°	0.006					
		90°					0.005	0.012
	MF <0.001	10°	<0.001					
		20°	<0.001	<0.001				
		30°	<0.001	0.001				
		45°	<0.001	<0.001				
		60°	<0.001	0.014				
		90°	<0.001	<0.001				
	LO <0.001	10°						
		20°		0.005				
		30°		0.011				
		45°		<0.001				
		60°						
		90°	0.005	<0.001		0.050		0.046

For the opposite force directions only the relative amplitude differences of the rectus abdominis muscle (RA) were not affected by the load level. Anyhow, both oblique abdominal muscles revealed only spare significant results within the multiple comparisons. Contrasts for the back muscles systematically occurred mainly at low load levels (MF: 5 and 10° vs. all other angles, LO 5 vs. 90°; 10 vs. 20°, 30, 45, and 90°; 30 vs. 90°; and 60 vs. 90°).

### Backward tilt

**Figure 2.**
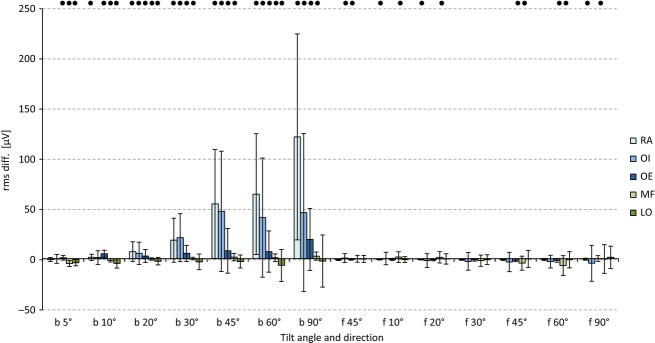
Differences in the SEMG amplitudes for posture‐controlled versus force‐controlled situations: positive values indicate larger amplitudes for posture‐controlled situations. Significant differences between both situations are marked with black dots. Values are displayed as the mean values ± standard deviations. b: backward tilt directions, f: forward tilt directions.

All monitored abdominal muscles demonstrated significantly higher RMS amplitudes in the posture‐controlled conditions than under force‐controlled conditions (RA: 10–90°, internal oblique muscle (OI): 20–90°, OE: 5–90°). The RA in particular, compared to the other abdominal muscles, and with increasing stress, had an over‐proportional increase in the RMS differences under the conditions of posture control (Fig. 2). In contrast, the detected absolute and relative SEMG amplitude differences for both back muscles, although statistically significant for almost every load level, were either of negligible amounts or even negative (i.e., lower amplitudes under posture control).

**Figure 3.**
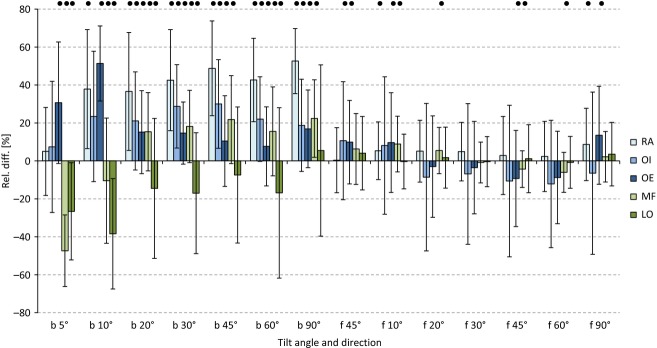
Relative differences in the SEMG amplitudes for posture‐controlled vs. force‐controlled situations: positive values indicate larger amplitudes for posture‐controlled situations. Significant differences between both situations are marked with black dots. Values are displayed as the mean values ± standard deviations. b: backward tilt directions, f: forward tilt directions.

The mean relative differences reached levels approximately 40% for RA, 22% for OI, and 20% for OE (Fig. 3). To test whether these findings were dependent on the load level, the corresponding relative differences were evaluated. According to the ANOVA results, the abdominal muscles' relative differences generally depended on the load level (Table 4). Post hoc tests indicated no systematic load dependency for the OI. All other differences could only be established at low tilt angles (RA: 5°, OE: 5°, 10°, MF: 5°, 10°, and LO: 10°).

Questioning of the subjects served as a subjective comparator of the extent of effort associated with the different control strategies. Higher effort was reported by 22 subjects for the posture‐controlled conditions, and 15 subjects reported higher effort for the force‐controlled conditions. Two subjects did not answer the questions. Consequently, in accordance with the perceived effort, no significant differences were detected (sign‐test: *P* = 0.324).

### Forward tilt

No systematic change in amplitude was detected for most investigated trunk muscles. The MF showed small but systematic differences caused by the change of the control strategy, whereby higher amplitudes of the RMS data were observed under posture control (20, 45, and 60°, Fig. 2).

Twenty‐four subjects rated the forward tilt under force control as more exhausting, and 10 subjects perceived posture‐controlled conditions as more demanding. Five participants did not answer the questions. Accordingly, the effort under force control was rated as significantly more strenuous (sign‐test: *P* < 0.05).

## Discussion

For the flexion tasks under posture‐controlled conditions, all studied abdominal muscles exhibited significantly higher RMS amplitudes than were observed under force‐controlled conditions (except for a few unsystematic results at low load levels). However, the control strategy seemed to have no relevant influence on the SEMG amplitudes of the back muscles. All detected differences in the antagonistic activity according to the respective force directions (i.e., back muscles under a backward tilt and abdominal muscles under a forward tilt) were very small, indicating that they are unlikely to be relevant.

### Inter‐muscular coordination

Given that the abdominal muscles' stress levels increase under posture‐controlled conditions, a rather complex argumentation is required, as one would expect that in the absence of fatigue, increased muscular stress goes hand‐in‐hand with an increase in force output. However, this assumption only appears to be true when a *single* muscle is investigated – when several muscles are interacting, further aspects need consideration. For instance, the relationship between the abdominal muscle activation patterns and their functional output cannot simply be derived from a limb muscle's performance. Any increase in activation within a limb muscle will lead to motion *or* force augmentation under isometric conditions. Consequently, to maintain isometric conditions, any force increase must be counteracted by antagonistic forces. In this context, antagonistic co‐contractions as well as increased external force levels can provide the required counterforce. Unlike a limb muscle, the abdominal muscles do not span between a single limb joint, but several intervertebral joints, and also retain the abdominal organs within the abdomen. Furthermore, they provide an essential counterforce for the back muscles to ensure spinal stability by controlling the intra‐abdominal pressure (Hemborg et al. 1985). Consequently, the abdominal muscles can be activated to a large extent without generating any visible motion, noticeable external effect, or even an increase in the back muscles' activity (Cholewicki et al. 1999). The enormous involuntarily evoked activity during lifting (Hemborg et al. 1985) and the voluntary increase in intra‐abdominal pressure (Thompson et al. 2006) during defecation or childbirth may be well‐elaborated examples of this. In other words, certain conditions provoke a considerable amount of abdominal activation without causing any biomechanical force.

One would expect the intra‐abdominal pressure to increase when, at a 90° backward tilt, the entire upper body weight is applied as the force moment and the subjects stabilize their upper body against gravity (position control, 90° backward tilt). However, this assumption cannot explain the approximately equal relative differences found at low tilt angles (Fig. 3) unless the posture‐controlled tasks generally provoke changes within the abdominal muscular activation patterns and therefore require an increase in intra‐abdominal pressure.

To fully explain the observed differences within the abdominal muscular activity, we would have had to investigate every single interacting trunk muscle. Unfortunately, our study only contains data for three abdominal and two back muscles. In particular, the latissimus muscle was not analyzed, and the data concerning the diaphragmatic or even pelvic floor activity (Thompson et al. 2006) cannot be provided. Importantly, phrenic activity correlates strongly with the intra‐abdominal pressure and could therefore explain our findings (Thompson et al. 2006). In summary, we cannot provide conclusive information regarding the influence of control strategies on the intra‐abdominal pressure levels.

Concerning the co‐contractions, the multifidus muscle revealed a significant difference between the posture‐ and force‐control due to a small but systematic increase in the SEMG activity under posture‐controlled load conditions. However, because the back muscles are superior to the abdominal muscles in terms of force, small changes in the electromyographical activity might result in higher force output (Ward et al. 2009).

**Figure 4.**
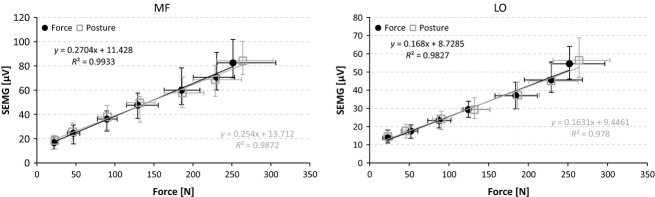
The amplitude–force relationship for the MF and LO muscles during forward tilt directions of force‐ and posture‐controlled tasks. Values are displayed as the median values ± quartile ranges. Equations for the linearly interpolated slopes are given together with their *r*² values.

To estimate the resulting counterforce, we calculated the amplitude over force relationship for the back muscles on the basis of both forward tilt conditions (Fig. 4). The two curves match almost perfectly, which is unsurprising because the fact that the back muscles have significant relative differences between the posture and force control has never been an issue (Fig. 3). In addition, the data confirmed the well‐known linear amplitude–force relationship for both back muscles (Anders et al. 2008). Based on this, we calculated the SEMG over force characteristics for both backward tilt situations for each investigated back muscle (Knutson et al. 1994). The calculation resulted in an average difference of 16 N for MF and 12 N for LO at 90° tilt angles in favor of the posture‐controlled tasks. Although the highest obtained values were considered, none were large and thus cannot explain the detected differences.

However, we cannot exclude deep multifidus participation because deep and superficial activation were demonstrated to be different from one another (Moseley et al. 2002). On the other hand, due to the muscles' close position to the spine, deep multifidus activity is more likely to enhance local stability than to exert relevant extensional forces. Thus, if deep portions of the multifidus muscle increase in activity under a backward tilt they would consequently provide increased spinal stability (Cholewicki and McGill 1995), which should lessen the abdominal activation.

### Interrelationship of technical and physiological factors

The aim of our study was to determine the effect of control strategies on the activity patterns of the trunk muscles. We had to rule out that our results might have been distorted by interrelated technical and physiological factors (Farina et al. 2002).

In any study investigating a predetermined sequence of tasks over a prolonged time, the SEMG amplitudes will be distorted by muscular fatigue (Merletti et al. 1990; Luttmann et al. 1996). Because the abdominal muscles are predominantly comprised of fast‐twitch fibers (Haggmark and Thorstensson 1979), the impact on them will be much greater than for the back muscles (Johnson et al. 1973; Rantanen et al. 1994; Mannion 1999). Nevertheless, given the individually randomized designs of studies and the short temporal duration of the load, the systematic influence muscle fatigue on our study results can be excluded.

Another technical issue refers to the SEMG signal: SEMG is subject to a large interindividual variability (Farina et al. 2004), typically requiring normalization (Lehman and McGill 1999). In this study we analyzed data on an individual basis, applying a repeated measures design. In this context, any additional normalization toward a reference situation (Lehman and McGill 1999) would have added the same offset, not influencing the results of any type. To account for interindividual amplitude differences we normalized the data according to the corresponding load situations by calculating the relative differences.

Variations in the SEMG sensitivity under muscular motion, that is, changes in posture, illustrate another technical factor worth considering (Farina et al. 2002; Mesin et al. 2009). We had to rule out the possibility that our results might have been distorted by changes in posture. In particular, the isometric contractions under the position‐controlled back‐tilt could have led to a flexion posture with the abdominal muscle shortening under high levels of stress. The forward tilt, on the other hand, was not subject to the risk of being affected by different body positions. Because of the identical relative differences between the control strategies (Fig. 3), which appeared both at the low and high tilt angles, this source of error could be excluded.

While efforts at tilt angles of 20° always achieved consistent results, the 5 to 10° tilt values contained outliers, requiring special attention. The lower stress level of the trunk muscles implicated a high variability in the execution accuracy, whereby no systematic difference between the control strategies could be determined. Moreover, with the low amplitude values observed, even small deviations of the RMS amplitude lead to large changes in the relative values (Fig. 3). Only forces above 20° showed relevant stress on the trunk muscles; therefore, this must be considered when evaluating the results.

Aside from that, the extension forces of the back muscles are vastly superior to the abdominals' maximum flexion potential in terms of force. However, assuming that the increased stress of the abdominal muscles from the postural control is caused by their comparatively lower force capacity, the relative deviation of the amplitude should increase with increasing tilt angles. However, even at a low load (20° tilt), the relative differences reached the same level as under the maximum load (90°, Fig. 3). Therefore, the different force‐generating capacities of the abdominal and back muscles alone cannot be responsible for the observed differences.

Thus, we can dismiss the influence of technical and physiological issues on the results of our study.

### Evolutionary biology

Last but not least, in terms of evolutionary biology, because of our upright body position the back muscles developed strongly in paravertebral orientation to ensure the necessary erection and stability of the spine. Likewise, load conditions that typically take place in front of the body promote the use of extension movement. This results in a daily force vector to the front that must be compensated from behind by the back muscles. Thus, the abdominals' force‐performance is therefore inferior to that of the back muscles.

It seems likely that human evolution from quadrupedal to bipedal mammals has not required any adaptation regarding the abdominal muscles. Thus, their function might still primarily consist of retaining the abdominal organs within the abdomen and providing an essential counterforce for spinal stability by controlling the intra‐abdominal pressure. Therefore, our findings might also indicate that the abdominal muscles rely on only a single “program” concerning motor control strategies, namely force control. As soon as they have to cope with something other than force‐controlled conditions, the necessary effort is overly increased.

## Conclusions

According to the present results, the back muscles can be used universally under the aspect of control strategies. Their muscular stress level shows no significant differences between posture and force control, which is why training can be performed without consideration of the control strategy.

Training‐oriented exercises, such as sit‐ups, are associated with significantly higher effort because they mainly require posture‐controlled contractions. To build the abdominal muscles gently while keeping the stress level to a minimum, force‐controlled efforts are thus particularly suitable. These findings could increasingly have applications in the context of rehabilitation.

## Acknowledgments

The authors thank Mrs. Elke Mey for technical assistance and the students of the Friedrich‐Schiller‐University who offered their time to take part in this study. This manuscript was edited for English language by Elsevier Webshop Support (ELS).

## Conflict of interest

We certify that no third party having a direct interest in the results of the research supporting this article has or will confer a benefit on us or on any organization with which we are associated.
